# Two stage hybrid approach for complex aortic coarctation repair

**DOI:** 10.1186/1749-8090-4-10

**Published:** 2009-02-24

**Authors:** Efstratios Koletsis, Stella Ekonomidis, Nikolaos Panagopoulos, George Tsaousis, James Crockett, Matthew Panagiotou

**Affiliations:** 1 Department of Cardiothoracic Surgery, School of Medicine, University of Patras, Patras, Greece; 2 Cardiac Surgery Department, Athens Medical Center, Athens, Greece; 3 Department of Paediatric Interventional Cardiology, Athens Medical Center, Athens, Greece

## Abstract

**Background:**

Management of an adult patient with aortic coarctation and an associated cardiac pathology poses a great surgical challenge since there are no standard guidelines for the therapy of such complex pathology. Debate exists not only on which lesion should be corrected first, but also upon the type and timing of the procedure. Surgery can be one- or two-staged. Both of these strategies are accomplice with elevate morbidity and mortality.

**Case report:**

In the face of such an extended surgical approach, balloon dilatation seems preferable for treatment of severe aortic coarctation.

We present an adult male patient with aortic coarctation combined with ascending aorta aneurysm and concomitant aortic valve regurgitation. The aortic coarctation was corrected first, using percutaneous balloon dilatation; and in a second stage the aortic regurgitation and ascending aorta aneurysm was treated by Bentall procedure. The patients' postoperative period was uneventful. Three years after the operation he continues to do well.

## Background

Coarctation of the aorta is a common congenital defect which unless primarily detected and surgically corrected in childhood, may be undiagnosed until adulthood; where most patients discovered during investigation of systemic hypertension [1]. Moreover coarctation is associated with congenital or acquired cardiac pathology where surgical intervention is mandatory [[1,2], Additional file 1]. Management of an adult patient with aortic coarctation and an associated cardiac pathology poses a great surgical challenge since there are no standard guidelines for the therapy of such complex pathology. Debate exists not only on which lesion should be corrected first, but also upon the type and timing of the procedure. Surgery can be one- or two-staged. Although the one stage approach can sometimes be accomplished through a single incision involves complex surgical procedures [3-7], the two-stage approach implicates two operations performed throw median sternotomy and posterolateral thoracotomy [8-10] both of these strategies is accomplice with elevate morbidity and mortality.

In the face of such an extended surgical approach, balloon dilatation seems preferable as it is a less invasive and safer method for treatment of severe aortic coarctation [11].

We present herein an adult male patient with coarctation of the aorta combined with aneurysmal dilatation of the ascending aorta and concomitant aortic valve regurgitation. The aortic coarctation was corrected first using percutaneous balloon dilatation; and in a second stage the aortic regurgitation was treated using the Bentall procedure. The patients' postoperative period was uneventful. Three years after the operation he continues to do well.

## Case report

**Figure 1:**
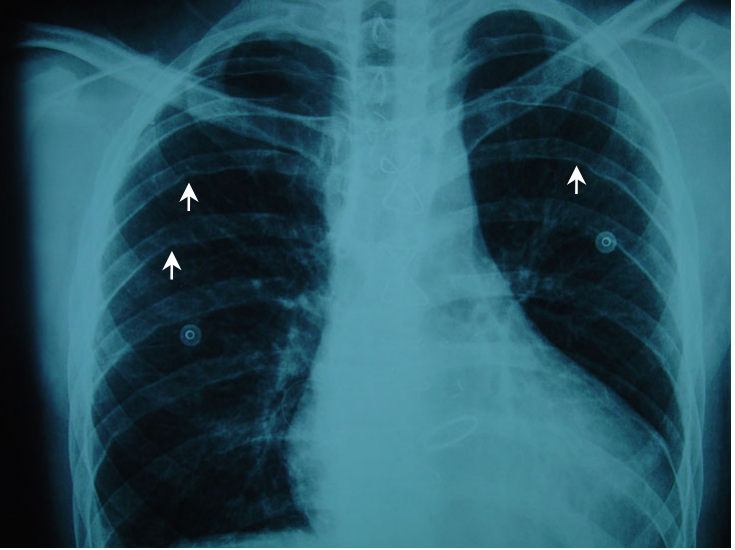
**Chest X-ray showed rib notching on the undersurface of the posterior ribs (white arrows)**.

**Figure 2:**
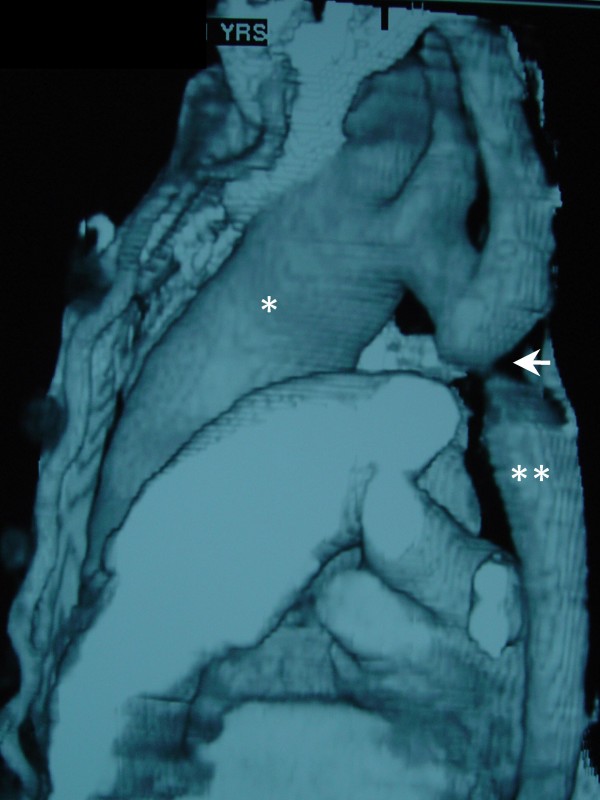
**Thoracic CT scanning (reconstruction) showing the coarctation site (white arrow)**. The asterisk depicts the aneurysmal dilatation of the ascending aorta which is of greater diameter compared to the descending thoracic aorta (double asterisk).

A 22-years old male patient presented with dyspnoea, fatigue and systemic hypertension. Physical examination revealed an increased second heart sound with an associated gallop rhythm and a diastolic murmur heard best at the cardiac apex. Radial pulses were normal but the femoral pulses were weak. Blood pressure measured at the left arm was higher than the one measured at the left leg. Chest X-ray showed rib notching and cardiomegaly (Figure 1). Further evaluation with echocardiography revealed an aortic root dilatation of 42 mm with ascending aorta dilatation 68 mm and concomitant severe aortic valve insufficiency (3+). The left ventricle was dilated with end diastolic diameter 75 mm and associated systolic dysfunction with an ejection fraction of 35 percent. Pulmonary hypertension 60 to 25 mean 40 mm Hg was also present. Thoracic CT scanning with intravenous contrast medium showed an ascending aorta aneurysm of 68 mm (Figure 2).

Cardiac catheterization was performed showing normal coronary arteries and severe aortic coarctation with a 70 mm Hg gradient measured across the descending aorta. The minimum diameter of the stenotic region on the aorta was 2 mm. The patient was classified as functional class III according to NYHA.

Correction of aortic coarctation was performed first by using a less invasive method. After heparinization and administration of antibiotic prophylaxis, dilatation across the coarctation site with balloon angioplasty was performed. Dilatation was accomplished by using 3 consecutive catheters of increasing size (8, 12, and 15 mm in diameter respectively). Post-dilatation aortography showed no residual gradients and the stenotic region was increased to 15 mm in diameter. The procedure was uncomplicated and the patient was released the following day.

**Figure 3:**
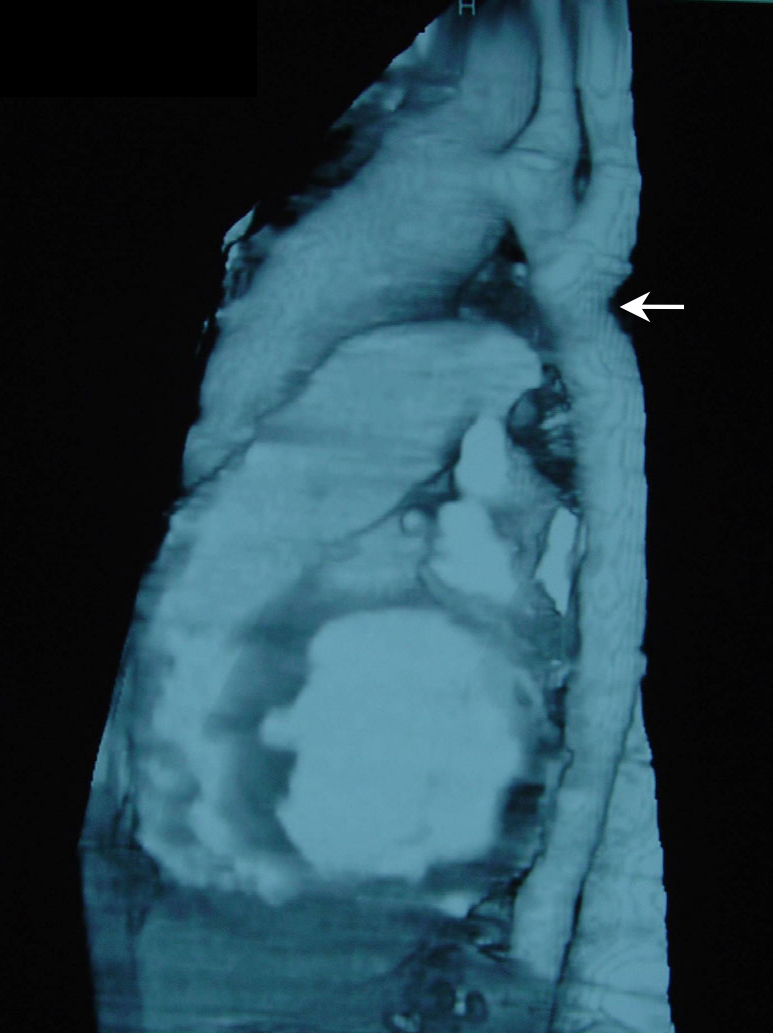
**Thoracic CT scanning 3 weeks after balloon angioplasty showing patency of descending thoracic aorta with no post-dilatational aneurysm formation (white arrow)**.

Two weeks after balloon angioplasty the patient was re-admitted for management of the intracardiac pathology. Pre-operational thoracic CT scanning was performed to exclude any post-dilatational aneurysm formation at the site of balloon anchorage and determine descending aorta diameter (Figure 3). Arterial cannulation at the right axillary artery was performed using a synthetic PTFE 8 mm graft. The purpose of this was twofold; to avoid the cannulation difficulties of inserting the aortic cannula into the hypoplastic femoral arteries and prevent vascular damage to the aorta after the recent angioplasty. A two-staged venous cannula was inserted into the inferior vena cava and cardiopulmonary bypass was established. Because of the ascending aorta aneurysmal dilatation and the concomitant aortic valvular regurgitation the patient underwent replacement of the ascending aorta with a metallic valved conduit (St Jude 27 mm) using the modified Bentall procedure. Cross-clamping time was 111 min and total bypass time was 138 min. The patients' recovery was unremarkable and he was discharged on the 12^th ^postoperative day.

At follow-up three years postoperatively the patient is normotensive with a functional aortic valve. Repeat CT scanning showed no re-coarctation or aortic aneurysm formation.

## Discussion

Coarctation of the aorta is a common congenital defect whose clinical manifestations become apparent during childhood. A considerable number of patients remain asymptomatic until adulthood where coarctation may be discovered incidentally when investigated for systemic hypertension [1]. With increasing age, the incidence among the patient population decreases but still remains significant. Liberthson et al. reported that 10.3% of patients (24 out of 234) presented with coarctation of the aorta after the age of 40 [12].

Management of aortic coarctation may represent a single surgical entity, or may be associated in a more complex form, with congenital or acquired intracardiac pathology where additional surgical intervention is mandatory [1,2]. The incidence of associated cardiac defects is greater in patients presenting after the age of 30 years (40%) than in those presenting in a younger age (27%) [12]. Moreover, 5 to 30% of patients with previous coarctation repair may require re-intervention [3].

Since the first operation for coarctation repair in 1944 [13], many aspects concerning the optimum surgical approach, the timing of surgery and the management of postoperative complications have been reconsidered. Additionally in cases with associated intracardiac defects, debate still exists on which lesion should be corrected first. These cases pose a great surgical challenge since no standard guidelines in the management of such complex pathology exists.

Complex forms of coarctation have been managed by anatomic and extra-anatomic bypass techniques. Complications of anatomic repair include intraoperative hemorrhage, recurrent laryngeal or phrenic nerve damage, chylothorax and intrathoracic sepsis [3,14,15]. Paraplegia due to spinal cord ischemia remains the most important complication [15,16]. Various extra-anatomic bypass techniques have been employed throughout the years for management of complex or isolated forms of coarctation with or without establishment of cardiopulmonary bypass [3-8,15].

For the correction of complex forms of coarctation different techniques have been employed. In the one-stage repair, simultaneous correction of both lesions can be achieved through a clamshell incision, a median sternotomy [3,5-7,17], a lateral thoracotomy [18] or by a combination of both incisions [8,19]. The two-stage repair can be performed through a combination of median sternotomy and lateral thoracotomy [8-10].

Another important question that needs to be answered by the surgeon is which lesion should be corrected first? The intracardiac lesion or the stenotic aorta?

Pethig et al. [17] reported heart failure and life-threatening ventricular arrhythmias in patients with one-stage aortic valve repair and consequent extra-anatomic bypass grafting for aortic coarctation. These were due to global myocardial ischemia and impaired coronary blood supply in hypertrophied hearts with low perfusion pressure. Furthermore, operating first on the cardiac defect may cause significant hypoperfusion of the organs distal to the stenotic region [20]. On the other hand, other surgeons did not observe any of these phenomena when using the one-stage approach, operating first on the intracardiac defect and subsequently performing the extra-anatomic bypass; thus avoiding sudden changes in coronary flow due to decrease in systemic vascular resistance when the coarctation is corrected first [3,5-7,11]. Therefore advantages of the one-stage approach, such as number of surgical procedures and decreased hospital stay, do not outweigh the significant risk of myocardial hypoperfusion and afterload reduction.

Avoidance of these phenomena can be achieved through the two-stage repair as mentioned earlier. On the other hand, valve replacement performed first as part of a staged procedure in a patient with left heart obstruction may result in difficulties coming off bypass and renal hypoperfusion [9].

A more conservative approach for coarctation repair may be of significant value since evidence suggests that transcatheter treatment provides an effective and safe alternative to surgical management [11]. Our patient presented with systolic dysfunction, left ventricular dilation and associated pulmonary hypertension. In the face of such pathology the one-stage repair may have resulted in hemodynamic instability. Therefore a two-staged approach was chosen. During the first step, repair of aortic coarctation through a less invasive method using balloon dilatation angioplasty was used. In this manner the patient may benefit with afterload reduction of a dysfunctional left ventricle; thus avoiding the increased hospital mortality and postoperative hemorrhage as well as minimizing devastating surgical complications. Additional maneuvers such as endovascular stent placement were considered unnecessary because: 1) the transluminal aortic gradient immediately after balloon angioplasty was almost zero and following the algorithm proposed by Zabal et al. stenting is considered when balloon angioplasty fails to reduce the gradient less than 10 mmHg [21], 2) stenting carries a considerable risk of aortic malpositioning [22]; and 3) issues of somatic growth changes in the thoracic aortic diameter. During the second step, aneurysmal dilatation of the ascending aorta with concomitant aortic valve regurgitation was corrected through a median sternotomy performing a modified Bentall operation.

## Conclusion

The non surgical treatment of the aortic stenosis proved to offer a significant advantage, converting a complex and risky surgical procedure into one of common practice.

## Consent

Written informed consent was obtained from the patient for publication of this case report and accompanying images.

## Competing interests

The authors declare that they have no competing interests.

## Authors' contributions

All authors: 1) have made substantial contributions to conception and design, or acquisition of data, or analysis and interpretation of data; 2) have been involved in drafting the manuscript or revising it critically for important intellectual content; and 3) have given final approval of the version to be published.

## Supplementary Material

Additional file 1 **Intervention of aortic coartation.** Click here for file
